# The Review of Selected Non-Pneumatic Tires Properties—Load Carrying Mechanism, Structure of Non-Pneumatic Tires

**DOI:** 10.3390/ma18071566

**Published:** 2025-03-30

**Authors:** Marcin Żmuda, Jerzy Jackowski

**Affiliations:** Institute of Vehicles and Transport, Faculty of Mechanical Engineering, Military University of Technology, Street gen. Sylwestra Kaliskiego 2, 00-908 Warsaw, Poland; jerzy.jackowski@wat.edu.pl

**Keywords:** non-pneumatic tire, airless tire, load-carrying mechanism, NPT structure analysis, NPT materials

## Abstract

In recent years, non-pneumatic tires have been gaining popularity, which can be seen in the increase in research results and proposals from world-class tire manufacturers (mainly as technology demonstrators). The possibility of eliminating the need to maintain compressed air is a major factor in the development of non-pneumatic tires and their usage in vehicles. Articles and patents were reviewed in relation to the load transfer mechanism, the design of non-pneumatic tire components, and recommendations for materials. Non-pneumatic tire top loaders are a desirable type of this type of wheel compared to bottom loaders, because they transfer loads over a larger part of the wheel, which increases their load capacity. Most non-pneumatic tires consist of a rim, an elastic structure, and a shear beam/band with a tread. The rim is used to secure the elastic structure and can be fitted with vibration dampers in the form of circumferential rubber rings. The gradient elastic structure, in comparison with the homogeneous structure (same thickness or dimensions of the elements), allows the range of axle displacements to be adjusted to the desired level without the need to increase the size of the wheel, and also influences the change in the location of the maximum stresses. The shear beam/ band mimics the properties of compressed air used in pneumatic tires. The shear beam/ band made as a webbing geometry ensures uniform pressure in the contact patch. The reinforced composite shear beam/ band ensures adequate bending strength with low energy losses and a small thickness of the beam/ band. Materials commonly used in the tire industry are used as reinforcement for the shear beam/ band, which was illustrated by the results of our own research.

## 1. Introduction

The pneumatic tire is currently the most commonly used type of tire. Its invention had a significant impact on the vehicle’s driving characteristics. Its invention is attributed to R. W. Thomson, who was the first to obtain a patent for his invention (1847 United States Patent) [1]. The dynamic development of the automotive industry at the beginning of the 20th century contributed to the development of the related tire industry. At that time, pneumatic tires and wheel designs that did not require compressed gas to be kept at a specific pressure to ensure driving characteristics were developed in parallel. World Wars I and II and the associated economic crises contributed to the search for alternative, reliable, puncture-proof solutions for road wheels. Unfortunately, the non-pneumatic wheel designs of that time have not survived to this day.

The road wheel is an important part of the vehicle because it transfers forces and moments between the car and the road surface. It is also an important part of the vehicle’s active safety system [2]. Modern pneumatic tires are a technologically advanced product characterized by: low rolling resistance (which translates into low energy consumption of the vehicle), good grip on dry and wet surfaces, smoothing and absorbing unevenness and damping vibrations from the road surface, and low pressures in the contact zone. Achieving these functions requires maintaining compressed gas at a specific pressure. The value of the inflation pressure will affect, e.g., the shape of the tread face, the length and width of the contact patch, and rolling resistance. The need to maintain the appropriate air pressure in tires was noticed by the legislative body of the European Union (EU), which resulted in the fact that from November 2014 every car manufactured and intended for sale in the EU must be equipped with a tire pressure monitoring system (TPMS) [3].

Pneumatic tires are currently used in various vehicles that can move on non-deformable surfaces (e.g., asphalt roads) and deformable surfaces (e.g., dirt roads). They must therefore have the required resistance to punctures, cuts, or tears. Damage to a pneumatic tire and the associated lack of the required inflation pressure results in the vehicle being immobilized and causes economic losses. It is therefore desirable to design a wheel that has the properties of a pneumatic tire without the need to maintain the appropriate inflation pressure. Modern non-pneumatic tires (NPTs) seem to be an alternative to pneumatic tires. According to [4], NPTs are mechanical devices that allow the transfer of forces between the road surface and the vehicle without the need to maintain a gas or fluid at a specific pressure. The presented definition is the appropriate criterion for determining whether a given wheel/tire can be classified as an NPT. The mimic of the compressed air properties will be carried out through structural support and the use of appropriate materials that provide the desired elasticity and strength of the NPT. The lack of compressed air or other gas allows them to be used in unfavorable terrain conditions, e.g., construction sites, or in vehicles that require high tire reliability, e.g., military vehicles. There are review publications on NPT. For example in [5] an overview of NPT regarding the materials used, transfer of vertical and tangential forces, grounding characteristics, and forming technology is presented. The construction of the most common NPT structures is not analyzed in detail. The paper reviews and compares the mechanisms of vertical load transfer and the NPT structure. Much emphasis is placed on the analysis of patented solutions. Most of the presented information is based on the results from research articles, such as fine element analysis (FEA) simulations or experimental research, and patents that present potential commercial applications of NPT.

Section 2 presents the applied literature analysis methodology, which uses scientific publications and patents.

Section 3 presents the mechanism of vertical load carry used in NPT. The influence of the NPT elastic structure on its load-carrying capacity is analyzed. Two main mechanisms (top and bottom loader) were presented.

Section 4 presents a detailed analysis of the NPT structure. The types of rim, elastic structure, and shear beam/ band that are used in NPT are presented.

Section 5 presents patent-based information regarding recommendations for materials used for specific parts of the NPTs and some examples of commercially available materials.

## 2. Review Methodology

The literature review was divided into two stages. The first stage included the analysis of planetary applications, while the second stage included the analysis of scientific publications. “Google Patents” was used to analyze patent applications and the following search words were used: “non-pneumatic tire”, “non-pneumatic tyre”, “airless tire”, “airless tyre”, “shear beam”, and “shear band”. The lower and upper limits of the time period of the analyzed period were not specified. The solutions that could be used in the present were analyzed. During the analysis, the focus was on the method of making the wheel and connecting its individual components, the type of materials used, and the impact of the NPT’s construction on the basic operating parameters of the NPT. Patent analysis was conducted in August and September 2024.

**Figure 1:**
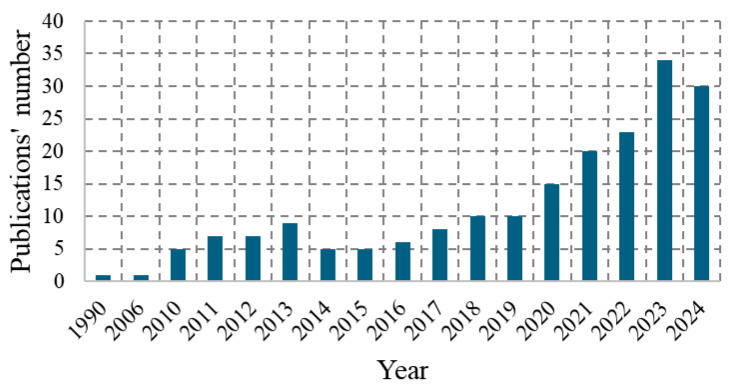
Number of publications on NPT in particular years.

The second stage included the analysis of the Scopus database. The following terms were used for the search “non AND pneumatic AND tire” OR “airless AND tire”. The following filters were applied: search period—up to 2024; language—English, keyword: “non-pneumatic tires”, “non-pneumatic tire”, “airless tires”, “non-pneumatic tyre”, “airless tire”, “nonpneumatic tire”, “non-pneumatic tire (NPT)”, “non-pneumatic wheels”, “non-pneumatic wheel”, “non-pneumatic tyres”, “NPT”. Figure 1 shows the number of Scopus publications for the adopted search criteria.

The publications were analyzed in terms of the vertical’s load-carrying mechanism, NPT structure, directional stiffness (radial, longitudinal, lateral), contact area, rolling resistance, and materials. The analysis omitted publications on aerodynamics, NPT heating, overcoming road irregularities, and fatigue life prediction. The analysis was conducted in October and November 2024. The article presents an analysis of the vertical load-carrying mechanism used in NPT and a detailed design of NPT components. The remaining analyzed areas will be published in another publication. This paper presents part of the literature review, which due to the large number of pages, will be presented in subsequent publications.

## 3. Vertical Load-Carrying Mechanism of NPTs

**Figure 2:**
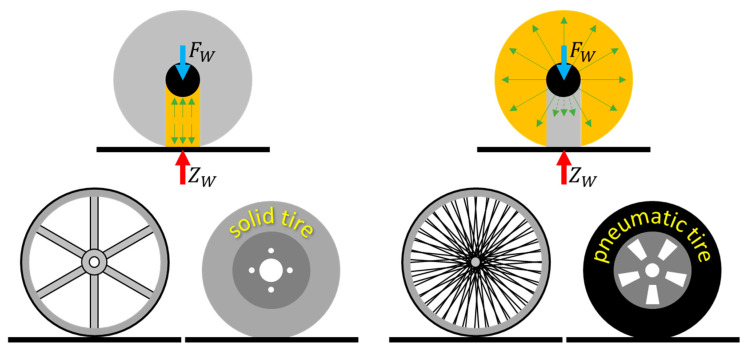
Carrying mechanism of vertical load, on left: bottom loader, top loader (figure based on [6]).

Modern wheels can be divided into two groups, depending on the way they transfer vertical loads related to the vehicle’s mass and the influence of road unevenness (Figure 2) [6,7,8,9,10,11,12,13]:

- bottom loader—the loads are carried by the part of the wheel located between the axle and the contact area,
- top loader—the loads are mainly carried by the part of the wheel that is outside the contact area.

Bottom loader wheels carry loads by compressing the part located between the contact area and the wheel axle. This type of mechanism requires appropriate strength of the material used, due to the fact that only a small part of the wheel carries the load at a given moment. In the case of carrying large loads, it is necessary to use wheels with large diameters or of considerable width, which influences an increase in their mass. In order to illustrate the bottom loader mechanism of the wheel, it is possible to make a mental division into 2 parts in the static state, i.e., the part that can be removed (because it does not carry loads) and the part that will carry loads to the road surface (located between the axle and the contact zone). This load-carrying mechanism will be characteristic of solid wheels and solid cushion tires.

The top loader wheel carries loads through the part located outside the contact zone as a result of the tension of the elements that are part of the main load-bearing structure. The top loader mechanism will be explained using the example of a spoked wheel and a pneumatic tire. The assembled spoked wheel is equipped with pre-tensioned spokes. In a pneumatic tire, the inextensible carcass threads, which will be subjected to the pressure of compressed air, will mimic the spokes mentioned above. Deformation of the top loaders (spoked wheel and the pneumatic tire) in the contact area causes a local reduction in the tension of load-bearing elements (the spokes, cord threads).

The vertical load is then transferred by the part of the spoked wheel or tire located outside the contact area. In the wheel part under the wheel axle, the loads are transferred to a lesser extent. Analyzing the spoke wheel in static conditions, the vertical load will be transferred to the surface despite the removal of the spoke located between the wheel axle and the contact zone, which illustrates the top loader mechanism. According to [8], the upper part of the NPT elastic structure can carry 90–100% of the vertical load, while the share of the lower part will be smaller, i.e., 0–10%. The main load on the NPT top loader spokes is tension and shear, not compression. This load transfer mechanism will also be characteristic of pneumatic tires.

When using the same wheel dimensions, the top loader mechanism will allow carried greater vertical load compared to the bottom loader. It is therefore desirable to use this mechanism in NPTs. Pneumatic tires have a number of advantages mentioned above. In the construction of modern NPTs, the advantages of pneumatic tires are attempted to be replicated by the proper selection of materials and the arrangement and shape of the NPTs components. With the increase in the hardness of the wheel materials, the vertical deflections of the wheel will decrease [9,14].

## 4. Construction of NPTs

**Figure 3:**
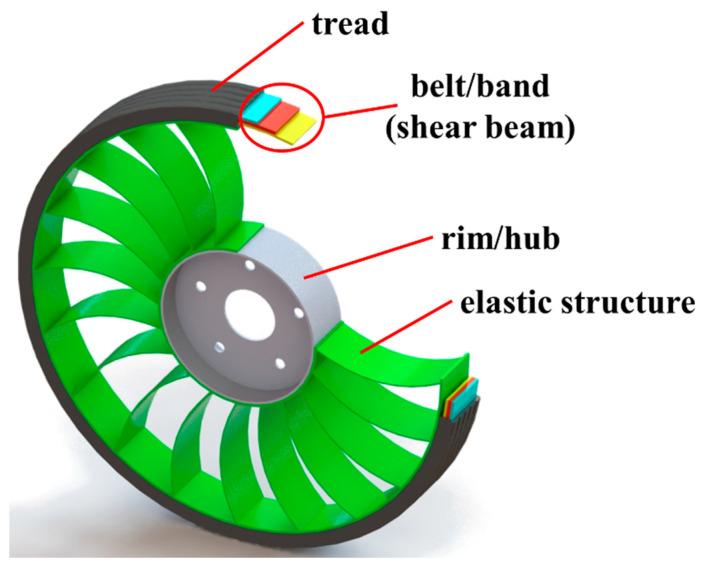
General construction of NPT.

The following components can be mentioned in the construction of modern NPTs (Figure 3) [15,16,17]:

- rim/hub,
- elastic structure,
- shear beam,
- tread.

### 4.1. Rim

The rim and wheel disc are designed to enable the connection of NPTs to the vehicle. Due to the often different constructions than in the case of pneumatic tires, this element is also called an NPT hub. The shape of the rim depends on the method of connecting to the NPT. The rim and wheel disc can be made of steel alloys or aluminum alloys as single-piece or multiple-piece rims. The following types and methods of connections can be used to connect the rim to the flexible structure [7,18,19,20]:

- adhesive connections (e.g., cyanoacrylate, polyurethane adhesives),
- mechanical and shape connections (e.g., screws, clamps, gaps, clamping bands), which provide, e.g., the appropriate pressure force on the rim,
- making an NPS’s elastic structure directly on the rim (which is one of the elements of the mold), e.g., by casting the polymer into the mold.

**Figure 4:**
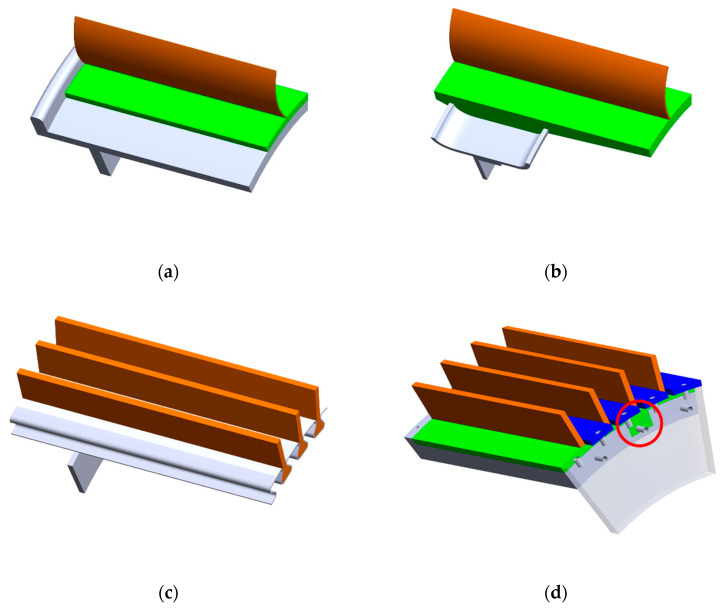

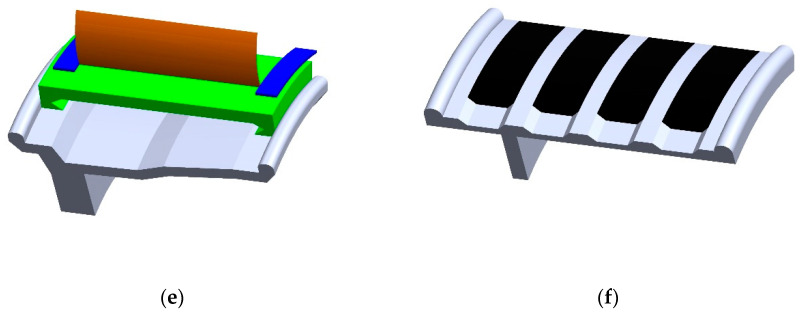
The shape of the rim for connection with the NPT’s elastic structure: (**a**) cylindrical shape, (**b**) U-shaped rim with a width smaller than the width of the NPT’s inner ring, (**c**) grooves made along the circumferential direction of the rim, (**d**) multi-piece rim with flat bars ensuring pressure of the NPT’s elastic structure to the rim, (**e**) use of a pneumatic tire rim, (**f**) additional rubber elements influencing the resultant stiffness of the NPT (figures based on [7,18,19,20]). Rim-grey, inner ring-green, elastic spokes-orange, vibration dampers-black, circumferential groove cooperated with the projection on the inner ring-red circle.

Figure 4 shows selected rim shape solutions. The rim and wheel disc are shown in grey color. The NPT’s elastic structure consisting of an annular inner circumferential element (so-called inner ring) and spokes are shown in green and orange colors, respectively. Elements providing pressure and additional elements made of elastomer are shown in blue and black colors, respectively.

The most common solution is to use a cylindrical rim (Figure 4a) with a one-sided flange, which allows for the placement of an elastic structure during the NPT assembly process. The elastic structure can then be connected to the rim using an adhesive connection. The use of a rim with a smaller width requires the production of a thicker NPT’s inner ring, which will partially take over the function of the wheel rim (Figure 4b). The connection of the rim with the elastic structure takes place, for example, during the casting of the elastomer into the mold used to produce the elastic structure. The presented solutions ensure good mutual connection and structural integrity. However, there is a problem with replacing worn or damaged NPT components, therefore solutions allowing the disconnecting of NPT components are sought. Figure 4c shows a wheel rim made of a single suitably shaped metal sheet that was welded to the wheel disc [7]. A rim with grooves made along its entire width along the circumferential direction mimics a splined connection. A similar analogy can be found in Figure 4d, where a multi-piece rim is shown. The rim is equipped on one side with a flange with circumferentially arranged threaded holes. On the circumference of the cylindrical rim (between the flange and wheel disc), several circumferential grooves are made (only one is visible in the drawing) cooperating with the projections on the inner ring. Connection marked by red circle. Pressure on NPT elements and rim is provided by flat bars (blue color), which are screwed to the flange and wheel disc. This type of connection, due to the greater number of elements, increases the resultant mass of the wheel and requires the use of an appropriate rim thickness to enable correct operation with the projections of the NPT’s elastic structure. The pressure of the elastic structure to the rim can also be ensured by circumferentially placed connectors (Figure 4e), made of metal or resin, ended with commonly known elements using to connection, e.g., screw connection [19]. The inner ring connected to the spokes is then divided into several segments of equal or different lengths (different lengths have a beneficial effect on damping vibrations during driving caused by the rotation of the tire—improving driving comfort). The connector can be placed on the circumference outside or inside (through a hole) of the split inner ring of the flexible structure. The use of the through hole prevents the displacement of the connecting element and ensures reliable attachment of the NPT to the rim. Similar functionality can be achieved by using a circumferential groove on the external attachment of the connector. In this connection method, the wheel rim used for pneumatic tires can be used, but this requires the production of a “bead” in the NPT’s inner ring.

During the NPT rotation vibrations may occur, the source of which will be the cyclic deformation of the elastic structure. Placing vibration dampers in an appropriately modified rim (Figure 4f) can reduce the aforementioned vibrations [20]. The modified rim contains circumferential grooves at fixed intervals, in which elastomeric (rubber) vibration dampers are placed. It is recommended that the distance between adjacent grooves be in the range of 30–50% of the groove width. Making the grooves at a distance smaller than recommended may result in a decrease in the effectiveness of the adhesive connection of the MNPT’s elastic structure to the rim, while increasing distance may reduce the vibration-damping effect. The recommended groove depth should be in the range of 20–50% of the wheel rim thickness. A greater depth value may negatively affect the strength or the weight of the rim, while a smaller depth may negatively affect the vibration-damping effect. The vibration isolator material should have an elastic modulus less than or equal to 20% of the material’s elastic modulus used on NPT’s elastic structure. The reduction in the vibration amplitude occurs as a result of the change in the equivalent stiffness of the entire wheel.

Quick summary:

- Based on the analysis of the literature, it was noticed that the issue of the rim is omitted in the articles.
- The cylindrical rim with a one-sided flange is the most common solution.
- The use of a mechanical connection to connect the elastic structure allows its replacement if necessary.
- Damping of vibrations associated with NPT movement can be successfully achieved by dampers placed around the circumference of the rim.

Future research of rim:

- Increased use of mechanical connections is expected due to the possibility of multiple uses of the rim.

### 4.2. NPT’s Elastic Structure

**Figure 5:**
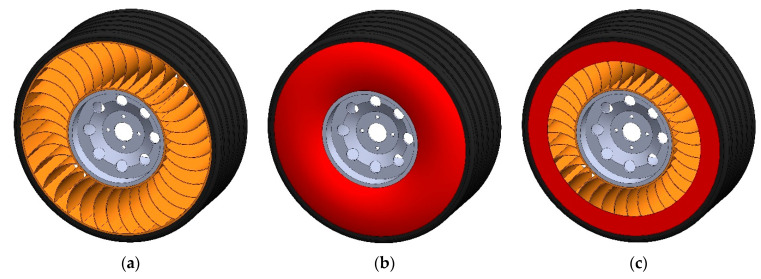
Type of side covering NPT’s elastic structure: (**a**) open, (**b**) closed, (**c**) mixed (figures based on [21,24]). Rim-grey, elastic structure-orange, shear beam with tread-black, side cover–red.

The elastic structure is the most characteristic element of NPTs and reflects the properties of compressed air in a pneumatic tire [15,19]. Its main task will be to provide an elastic-damping connection between the rim and the band with tread [21,22,23]. Depending on the method of its construction and connection with other elements, the NPT may be equipped with an inner and outer annular circumferential element, the so-called inner and outer ring (enabling connection with the rim and the NPT band). Taking into account the way of covering the NPT side, the following types of elastic structure can be distinguished [21,24,25] (Figure 5):

- open—empty spaces are visible in side view; the types that provides good cooling,
- closed—side covers (e.g., flat or domed rings) completely covering the flexible structure or filling the empty spaces with additional material,
- mixed—the NPT’s elastic structure is partially covered.

Side covers (Figure 5b) or material filling the open spaces, depending on the method of construction, can participate in carrying vertical loads.

The materials used and the arrangement and shape of the elastic structure will affect, i.e., the driving properties and the load-carrying mechanism, as well as the dimensions of the wheel [7]. The “density” of the NPT elastic structure will determine what loads its individual element is subjected to during the rotation of the wheel and in the side view can be described by the coefficient [15]:

(1) $$f_{A}=\frac{A_{OS}}{A_{T}}$$

- $f_{A}$—filling factor of the NPT’s elastic structure,
- $A_{OS}$—surface area of the open type elastic structure,
- $A_{T}$—total surface area of the elastic structure.

The elastic-damping properties and the shape of the NPT’s elastic structure will influence, i.e., the load-carrying mechanism, the range of NPT axle displacements, and the contact patch parameters (mainly the patch length) [6,26].

**Figure 6:**
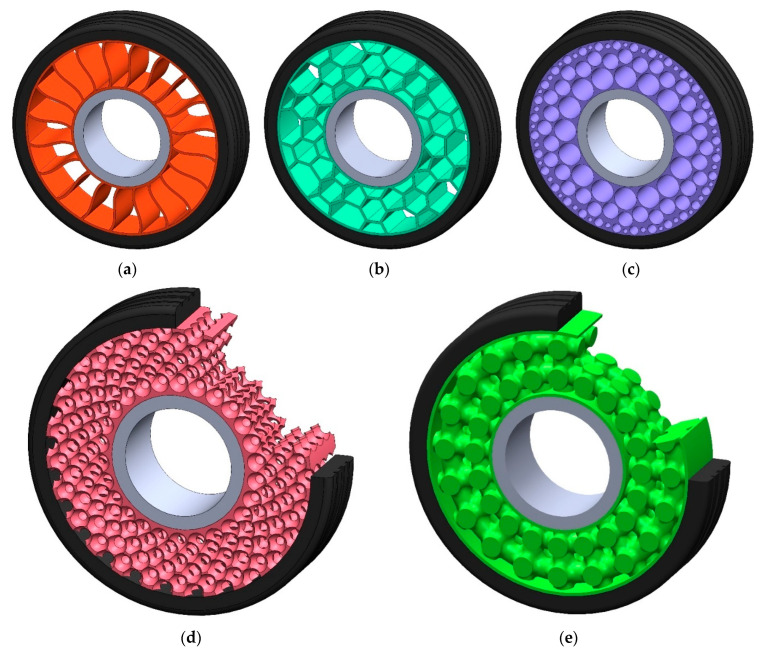
Types of elastic structure according to the way its elements are interconnected: (**a**) 2D spoke, (**b**) 2D cellular hexagonal honeycomb, (**c**) 2D cellular circular honeycomb, (**d**) spherical multi-hole, (**e**) rotated primitive type auxetic structure (figures based on [27,28,29,30]). Rim-grey, elastic structure-orange, shear beam with tread-black, side cover–red.

The elastic structure, due to the way its components are connected, can be divided into the following types [27,28,29,30]:

- 2D structure (Figure 6a–c)—an elastic structure formed by cutting or extruding flat geometric shapes in the axial direction of the NPT,
- 3D structure (Figure 6d,e)—individual elements of the elastic structure are interconnected in three-dimensional space, and the way of interconnecting is defined during engineering designing.

**Figure 7:**
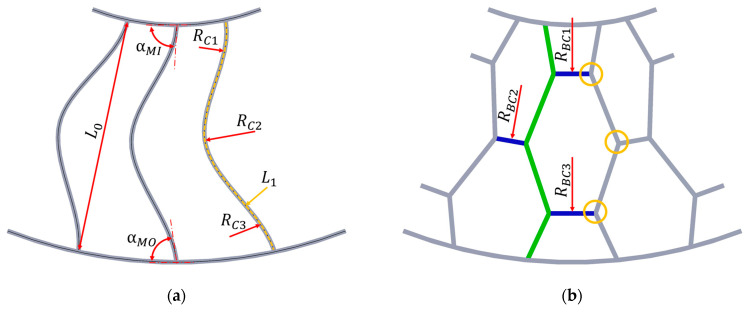
Types of elastic structure according to the shape of its elements: (**a**) single spokes, (**b**) cellular (figures based on [21,22,23,24]). *R_Ci_*—curvature radius of elastic spoke, *R_BCi_*—radius of the circles defining the boundaries of repeating cells in a given layer, *L*_0_—the shortest distance between the spoke attachment points, *L*_1_—the spoke length of the undeformed spoke, defined by the centre line, *α_MO_*—spoke attachment angle of spoke to the outer ring, *α_MI_*—spoke attachment angle of spoke to the inner ring.

NPT’s elastic structure due to the shape of its elements can be divided into the following types (Figure 7) [15,21,22,23,24,26]:

- single spokes—single elements arranged circumferentially between the rim and the band, the ends of which lie on straight lines passing through the axis of rotation or their ends are oriented with a certain offset,
- cellular/layered—a structure composed of single cells of repeating shapes (polygons) located on a given layer, the boundary of which is defined by a circle with radius $R_{BCi}$,
- mixed—a combination of the above types; or a structure in which the predominant type of elements cannot be specified.

NPT equipped with single spokes may be characterized by stress concentration in their attachment points, where stretching and torsion will occur. In order to reduce stress in the above-mentioned places, spokes with a curvature described by several radii ($R_{Ci}$) are used, although the radii do not have to be equal (Figure 7a), which ensures obtaining the appropriate value of the spoke attachment angles to the outer ring $\alpha_{MO}$ and inner ring $\alpha_{MI}$. The curvature of the spokes, which expresses their excess length, can be described by the equation [22]:

(2) $$ExcessLength=L_{E}=\left[\left(\frac{L_{0}}{L_{1}}\right)-1\right]\times 100\%\;$$

- $L_{0}$—the shortest distance between the spoke attachment points,
- $L_{1}$—the spoke length of the undeformed spoke, defined by the centre line (centre line—dashed orange line in Figure 7a).

Figure 7b shows a structure built of single cells, the arrangement of which is repeated on a given layer limited by a circle of radius $R_{BCi}$. Cells usually increase their dimensions as they are located farther from the NPT’s axis rotation. In a single cell, it is possible to specify sides arranged radially or at a certain angle to the radial direction (green) and sides (connector) arranged tangentially to the circles $R_{BCi}$ or at a certain angle to the tangential direction (blue). A cellular structure can be created as a result of expanding a structure built of single spokes with connectors tangent to the concentric circles, which is why this type of structure is also referred to as a lattice spoke [31]. A cellular structure is also made with a negative Poisson’s ratio. In such cases, cells are used in which a concave angle is applied between the selected sides [32,33]. The angle at which the sides of the polygons of the layered structure connect with the remaining NPT elements will affect the stress concentrations, similarly as described for the spoke structure. The stresses will decrease the closer the angle is to a right angle [32].

The desired top loader mechanism requires ensuring an appropriate susceptibility to buckling of the elastic structure between the NPT axis and the contact path. Susceptibility to bending and buckling during compression of individual spokes is obtained by providing an appropriate curvature, eccentricity of the attachment points, and their small thickness in comparison to their width and length [21,23,24]. The necessity of maintaining the circularity of the band and avoiding their mutual contact determines the number of possible individual spokes [14,23]. Susceptibility to buckling of the cellular structure can be obtained by using a local reduction in the thickness of the cell wall or by making each side of the cell with an appropriate curvature [34].

**Figure 8:**
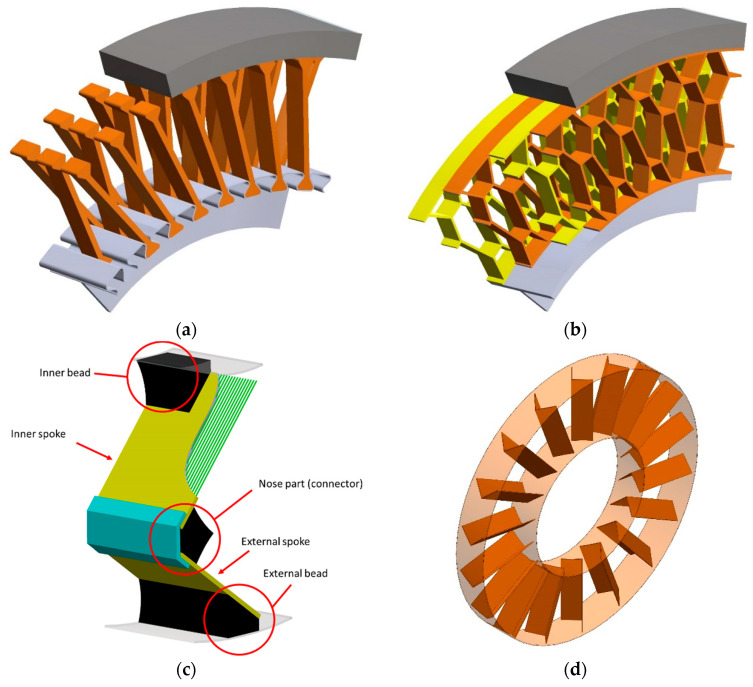

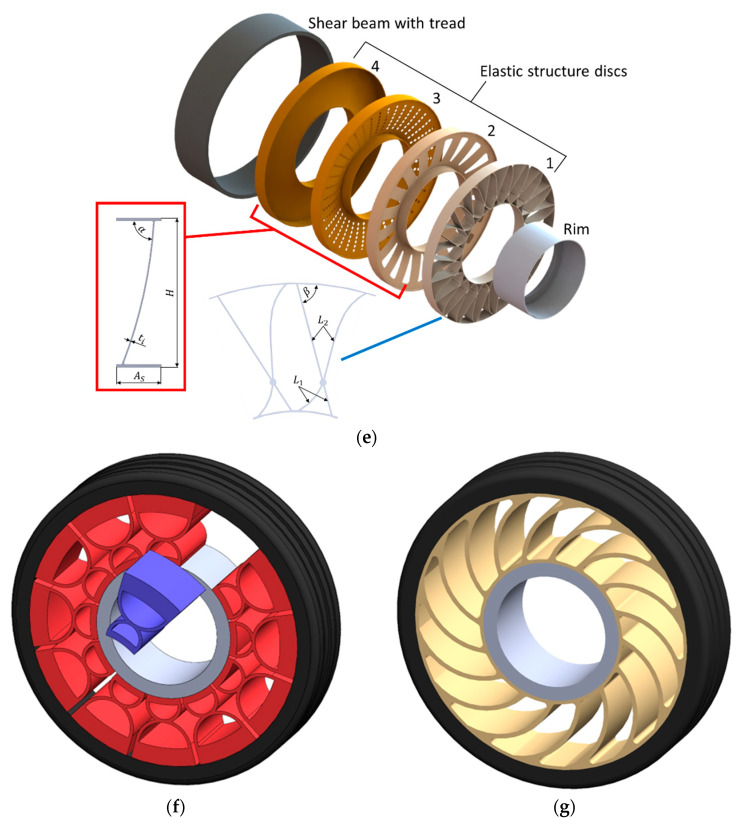
Examples of elastic structure shapes: (**a**) single spokes mechanically attached to the NPT rim, (**b**) cellular structure divided into axial segments, (**c**) composite spoke, (**d**) zig-zag type, (**e**) consisting of discs enabling the carry of tensile and shear loads, (**f**) radial segmented flexible structure (**g**) sloping spokes type (figures based on [7,8,9,11,12,23,34,35,36,37]).

Figure 8 shows selected solutions for the design of the NPT’s elastic structure and the methods used to connect it to the remaining NPT elements. The elastic structure equipped with an outer and inner ring is usually created by casting an elastomer into a mold. This method of production ensures the integrity of this part of the NPT [7]. On the other hand, integrity makes impossible the use of different materials and the use of a different shape of the spokes or the cellular structure than the shape defined by the mold. Casting is also not conducive to the use of reinforcements (e.g., cord fabrics) in the elastic structure. A solution may be to make the elastic structure in the form of individual elements, e.g., spokes mechanically attached to the wheel rim (Figure 8a). Such a solution allows the replacement of damaged elements and the possibility of using different materials.

Figure 8b shows a cellular structure divided into identical disc segments. Two groups of segments angularly shifted relative to each other are marked in different colors. All segments have the same dimensions of the elastic structure. The shift is intended to simplify the buckling of the structure under the NPT axle, improve the smoothing and absorption properties of ground unevenness, and reduce noise, as well as provide greater torsional stiffness [34]. The segmented structure of the elastic layer allows for easy shaping of the target characteristics of the wheel by changing the number of segments used.

An example of the possibilities of forming the NPT characteristics is shown in Figure 8e, whose elastic structure consists of at least one disc (1–4) [8]. Discs 2–4 transfer tensile and shear loads, while disc 1 can transfer only tensile loads (tensile loads -> top loader mechanism). Discs do not transfer compressive loads, they buckle under the NPT axis. Disc 1 will be characterized by high stiffness in the lateral direction and can be made of intersecting spokes, e.g., one of them is made with two curvatures, while the other is a straight line tangent to the outer ring at an angle β = 20 ÷ 80°. The intersection point in this example is determined by the length ratio $L_{1}/L_{2}$ and the most advantageous solution was obtained for values in the range of 0.3 ÷ 2. Discs 2–4 differ in the number and area of empty spaces. The spokes (disc 2) can have any cross-section, their susceptibility to buckling depends on the ratio of their width $W_{i}$ to the thickness $t_{i}$ (the best solution was obtained for $W_{i}/t_{i}$ = 45 ÷ 55). The shear load-bearing capacity of rectangular spokes (disc 2) is determined by the geometrical relationship $H/t_{i}$ and the best results are obtained for values in the range of 12 ÷ 17. The widths of the discs should be 5–20% of the NPT width and the width of each disc can be different. The type and number of discs used allow for forming the target directional stiffness characteristics of the NPT. In order to ensure the load-carrying mechanism of the elastic structure, it should be stiffer than the band. According to [8], it is advantageous for the radial stiffness of the segmented disc with spokes to be in the range of 4 to 12 times greater than the band stiffness.

Figure 8c shows the use of two composite spokes connected with an elastomer connector [35,36]. The V-shaped spoke has an inner bead attached to the wheel rim and an outer bead attached to the band. The composite spokes are connected to each other in the nose section using an elastomer and can be additionally reinforced with a material commonly used in the construction of pneumatic tires (Figure 8c, element with sky-blue color). The inner and outer spokes are made of a material with relatively greater bending stiffness than the elastomer, e.g., glass fiber-reinforced resin. Depending on the current position relative to the axis of rotation, the spokes will be compressed and stretched. The elastomer in the nose section will act as a hinge. The inner and outer spokes can have different lengths, which provides the necessary space for proper operation when the wheel is loaded. At the wheel design stage, the value of the vertical displacement of the wheel axis can be selected, which will cause the contact of the nose part with the outer bead of the adjacent spoke. The described cooperation will act as a bumper limiting excessive vertical displacements. The stiffness of the V-shaped spoke can be adjusted, i.e., based on the change in its length and the selection of materials for its production.

The arrangement of spokes made of elastomer in a “zig-zag” pattern (Figure 8d) ensures high directional strength while maintaining the ability to transfer high torque [23]. The spokes will be difficult to buckle and will cause pressure concentration in the contact zone where they connect with the shear beam and tread [9,37].

Figure 8f shows NPT, the elastic structure of which is made of segments [12], which are attached to the other parts of the NPT by chemical bonding (gluing). One of the segments is marked in purple for easier identification. The proposed elastic structure will work as a bottom loader. The advantage of the proposed solution is the possibility of selectively replacing damaged segments (selectively replace damaged support units). Individual segments can be treated as single spokes. The figure does not include the method of connecting the segments to the wheel rim proposed by the authors.

In [11] an elastic structure was presented (Figure 8g) which, for a stationary NPT loaded with a vertical force, caused a longitudinal force acting on the wheel axle. The elastic 2D structure was built of single spokes, which were made at an acute angle to the inner ring (sloping spokes).

In the design of the NPT elastic structure can be found solutions inspired by bionics, i.e., the possibility of using solutions from living organisms (plants and animals), as a result of evolution, in mechanical structures [38]. The aim of imitating nature’s structure is to reduce the volume of the material used and optimize the assumed criterion, e.g., increased strength.

The most common solution is the use of a honeycomb in a layered structure. The basic structure of a honeycomb consists of hexagonal cells with equal angles inside the cell. Honeycomb cells are characterized by high compressive strength and resistance to bending at a relatively low mass. The structure is space efficient because the small amount of material used results in a lightweight structure [39].

**Figure 9:**
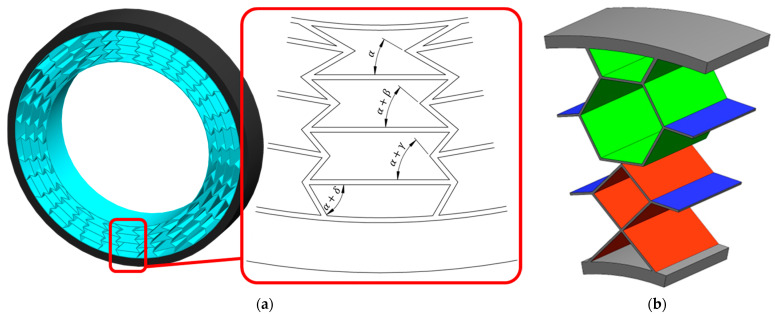
Examples of bionic, gradient and hybrid elastic structures: (**a**) honeycomb gradient structure with negative Poisson’s ratio, (**b**) hybrid elastic structure (rhombus-hexagon) (figures based on [32,40]). Repating hexagon shape-green, repating rhombus shape-red, connectors-blue.

One type of elastic structure is the gradient structure, which assumes the separation of sub-structures with different properties, which, when combined into a complete elastic structure, will ensure the achievement of specific functional requirements [32]. In gradient structures, the most common changes are the thickness of the elastic structure, the number of elements, and the change of internal angles (in the case of sub-cells of a honeycomb). Negative Poisson’s ratio cells are a modification of the gradient honeycomb structure (Figure 9a), which will work as an auxetic structure. The structure has been divided into sub-cells, the internal initial angle α of which is increased by a specific gradient (angles γ and δ may or may not be multiples of angle β).

The finite element research [32] have shown that increasing the base angle of the auxetic structure without intrducing a gradient causes a decrease in the vertical displacements of the wheel (increased radial stiffness). Small values of the internal initial angle α resulted in high stresses in the places where the structure is connected to the other parts of the NPT. However, taking into account the gradient allows for optimal shaping of the radial characteristic, reducing stresses in the places where the structure is connected to the outer and inner rings.

In [40], six types of elastic structures for NPT were analysed, including homogeneous (rhombus, hexagon, auxetic) and mixed/hybrid (rhombus-hexagon, hexagon-auxetic, and auxetic-rhombus) structures. The hybrid structure is a layered structure in which at least two layers of repeating cells, with a defined shape, can be distinguished (Figure 9b). Repeating shapes in the individual hexagon and rhombus layers are marked with green and red color, respectively. Connectors of adjacent cells are marked in blue color.

The analysed homogeneous structures were characterized by larger displacements of the wheel axis for a given load, which translated into shear beam deformation and contact area increase (contact pressure decrease). The results of the static property analysis showed that the rhombic-hexagonal hybrid structure had the most uniform pressure distribution on non-deformable ground.

The gradient cell elastic structure (modified honeycomb) was analysed in [41]. Changing the thickness of individual honeycomb walls affected the radial stiffness of the NPT. The radial characteristic of the NPT could be shaped by changing the thickness (in the range of 4 mm +/− 1 mm) of the honeycomb cell walls. Applying the same vertical load, the use of thicker walls farther from the wheel axis resulted in reduced displacements, while the use of thicker honeycomb walls closer to the wheel axis resulted in increased displacements.

Another example of the use of bionics can be found in [42], where bionic spokes described by curves inspired by petal dahlia flower petals were used to build a flexible gradient layered structure. The petal-shaped structure has high load carrying and stability. The disadvantage of this analyzed solution was high stresses at the connection point with the tread and rim due to the small angle between the spoke and the tangent from the connection point. The gradient included a smooth change in the spoke thickness and its curvature. By increasing the thickness of the elastic structures closer to the rim and reducing the curvature of the spoke, the radial stiffness of the Petal Bionic NPT was increased.

In [43], the vibration reduction mechanism of the cat’s paw pads, which was observed during the analysis of its movement, was used in the bionic design of the NPT spokes to solve the vibration problem (spokes with asymmetrical sidewall).

In [44] the lower limb of the kangaroo was inspired to design the NPT spoke with variable (gradient) thickness. The analyzed NPT was characterized by greater radial and lateral stiffness and lower circumferential stiffness compared to the analyzed pneumatic tire.

Based on the above information, the following NPT divisions can be proposed:

- according to the possibility of dividing the elastic structure:
  ○ uniform,
  ○ segmented (disc),
  ○ mixed,
- according to the method of connecting the elastic structure to the rim:
  ○ mechanical connection,
  ○ chemical connection,
  ○ mixed connection.

The use of elastomer as the material of the elastic structure ensures effective loads carried during the use of NPT in normal conditions and as a result of impact loads. The nonlinearity of the tensile elastomers characteristics allows the NPT to achieve high values of local deflections, e.g., as a result of the NPT driving over a significant road unevenness without generating high stresses. As a result, the elastic structure of the NPT will minimize impact loads transferred to the vehicle’s supporting structure [45,46].

Quick summary:

- The open, flexible structure facilitates NPT cooling but unfortunately is less resistant to external factors (e.g., rock, mud).
- The top loader elastic structure provides greater load capacity compared to the bottom loader.
- Adequate buckling resistance of the structure under the wheel axle is necessary to ensure under vertical load, which will reduce stress concentration.
- The elastic structure does not allow the elastic properties of NPT to change during exploitation, which distinguishes it from pneumatic tires (the range of vertical wheel displacement is shaped by the inflation pressure).
- The angle value at the connection points of the elastic structure to the rim and the shear beam has an influence on the stress level. The smallest stresses occur at an angle close to a right angle.
- The gradient cell/layer structure will change the location of maximum stresses, which allows for the optimization of the elastic structure.

Future research on elastic structure:

- The various types of elastic structures considered indicate that an optimal solution, that can be used in a wide range of vehicles (as is the case with pneumatic tires), has not yet been developed. A solution is expected whose basic design will provide the possibility of use in various types of wheeled vehicles.
- Nature, through evolution, provides optimal solutions, including optimizing the structure in terms of compressive or bending strength. Well-known cases are the use of honeycomb structure, and curves describing the shape of petals of some plants and animal limbs in the elastic structure of NPT. It is expected that the application of bionics in the elastic structure is an important and promising direction of NPT development also in relation to other wheel components.

### 4.3. Shear Beam and Tread

**Figure 10:**
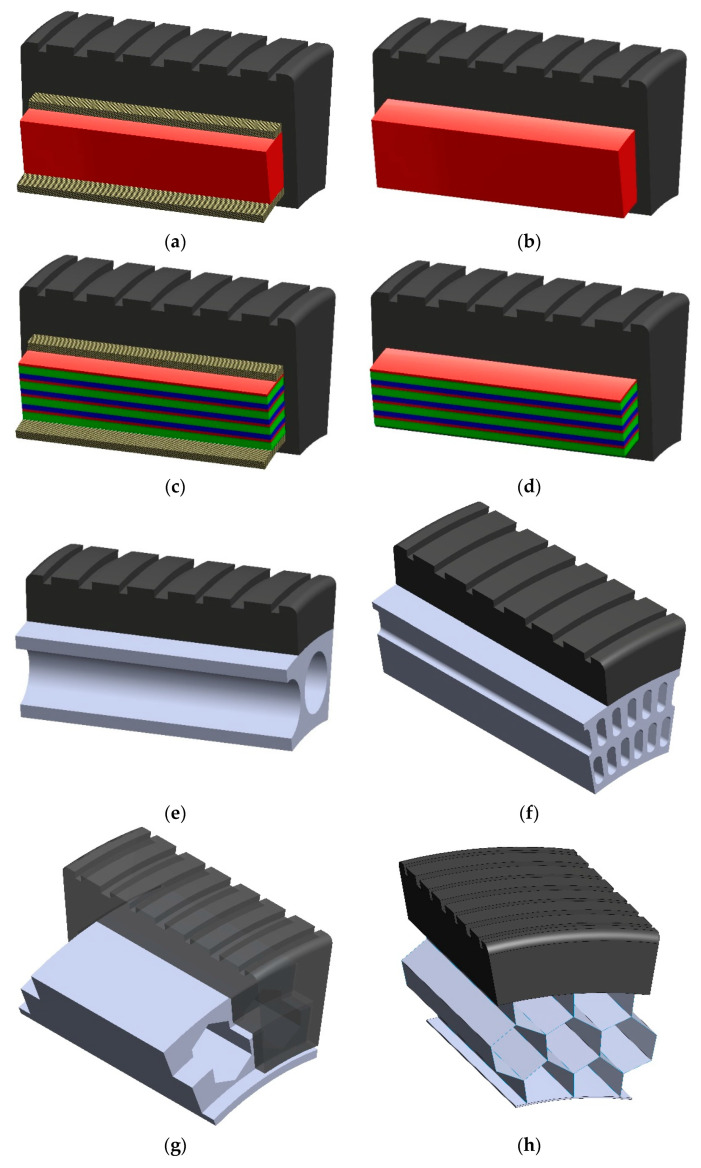
Shear beam/ band: (**a**) reinforced with a solid homogeneous core, (**b**) unreinforced solid homogeneous, (**c**) reinforced with a solid composite core, (**d**) unreinforced solid composite, (**e**) unreinforced homogeneous with through holes in one layer, (**f**) unreinforced homogeneous with through holes in two layers, (**g**) unreinforced with through holes covered by a tread, (**h**) made of non-elastomer, the geometry of which reflects hyperelastic properties (figures based on [24,45,46,47,48,49]).

The NPT shear beam (band) is a flexible ring, susceptible to bending, to which an NPT’s elastic structure is attached on the inside. On the outside of the shear beam is a tread, which can be made together with the shear beam or attached to it by a mechanical connection or chemical bond. The tread performs the same tasks as in a pneumatic tire. The following basic types of shear beams can be distinguished (Figure 10): reinforced and unreinforced [45]. The reinforced shear beam (Figure 10a,c) consists of at least one reinforcement layer of non-stretchable material, e.g., cord fabrics [6,8,23]. The cord threads, similarly to pneumatic tires, are arranged at a specific angle to the NPT rotation axis [8]. In the case of using more layers, the material (elastomer) located between the reinforcements will be referred to as the core. The reinforcement layers are located at specific distances from the wheel rotation axis and are intended to increase the resultant stiffness of the NPT [21,24]. Depending on the solution, the core can be made as a solid (Figure 10a), or composite (Figure 10c), or with a specific geometry. The solid core completely fills the space between the reinforcement layers and one (homogeneous core) or more (composite core) types of elastomer can be used to make it [47]. The unreinforced band (Figure 10b,d–g), in which no layers of non-stretchable material are used, is made using an isotropic or composite elastomer [45]. The lack of a band reinforcement layer does not exclude the possibility of its use in the tread.

Normal NPT load causes elastic deformation of the band, the part which under the wheel axle is flattened, shaping the contact area. In the case of a band consisting of two reinforcement layers and a solid core made of elastomer, the normal load causes a relative displacement of the reinforcement layers, which causes shear stresses in the core layer above the contact area (shear beam showed on Figure 10a). The described feature of the band is carry out by the properties of the materials used, i.e., the reinforcements have a longitudinal tensile modulus of elasticity sufficiently greater than the shear modulus of elasticity of the elastomeric shear layer [48].

The use of a solid composite core (Figure 10c), i.e., made of at least two uniform elastomer layers, results from the difficulty in finding a material that is simultaneously characterized by a high dynamic shear modulus G, high elongation at break and low energy losses during deformation (hysteresis) [47]. Fulfilling the first two requirements by a full homogeneous core would require increasing its thickness. The composite core allows for copying the parameters of a uniform core at the same time reducing its thickness. Composite core is made of a “soft” and “stiff” elastomer. The first of them (“soft”) is characterized by a lower dynamic shear modulus $G_{L}\;$and a low hysteresis coefficient at relatively high shear stress values. The “soft” elastomer improves the strength properties of the core at the cost of increasing its thickness. The second elastomer layer (“rigid”) is characterized by a higher dynamic shear modulus $G_{H}$ and a low value of energy losses (hysteresis coefficient) in the range of small shear stress deformations. This elastomer allows for a reduction in the core thickness, which in turn reduces its strength. A similar phenomenon is observed for the unreinforced composite band (Figure 10d). The lack of reinforcement layers will affect the reduction of shear deformations of the uniform band (Figure 10b), which in the NPT will partially mimic a single reinforcement layer. The advantage of not using reinforcement layers is the possibility of making the band using centrifugal casting, obtaining a uniform thickness of the solid band or individual layers of the composite shear beam.

The use of holes in the unreinforced band is intended to ensure susceptibility to shear deformation (Figure 10e–g). The band (Figure 10e) has a monolithic structure, which is made of a homogeneous material. In this band, two circumferential bands between which there is a webbing geometry can be distinguished. Webbing geometry band resulting from the removal of band material. As the distance between the band through holes decreases, the number of holes and the amount of material removed increases, which causes a decrease in the value of the effective shear modulus [39]. They can have any shape. In order to protect against the accumulation of e. g. mud or small rocks, the band holes are made as conical inwards [38]. Another example of protection is shown in Figure 10g. The band is covered with side tread flaps, providing mechanical protection [39]. In the band shown in Figure 10f, three circumferentially continuous bands and two web structures can be distinguished. This type of band will be mainly intended for NPT with a large external diameter and high load-carrying capacity. Making a larger number of layers of the web structure allows for the proper selection of the band stiffness by changing its geometry. Changes in stiffness can also be achieved by using, e.g., a composite structure or reinforcement layers, which, however, in comparison to the web structure, will be more difficult to perform during the production process [46]. The through holes of the homogeneous unreinforced band provide simplicity resulting from the use of a homogeneous material, without the need for reinforcements or a composite structure.

In [49] the possibility of using an aluminum alloy in a honeycomb band was analyzed. The geometrical dimensions (wall thickness; angle, height and width of the cell) were adjusted in order to obtain a shear modulus corresponding to the elastomer. In the numerical tests, a wheel equipped with straight spokes was loaded with a force applied to its axis, what is more the contact pressure and the band stresses were analyzed. A non-uniform pressure distribution was obtained, i.e., the pressures on the rigid road were transferred by the elements connecting the honeycomb cells with the inner and outer band rings. The maximum stress of the band cells was observed near the beginning and end of the contact area, where the shear effect is the greatest, resulting from the proximity of the undeformed and “flattened” band parts. A more uniform pressure in the contact area, with their concentration at the beginning and end of the path, was obtained for tests with a solid unreinforced core.

**Figure 11:**
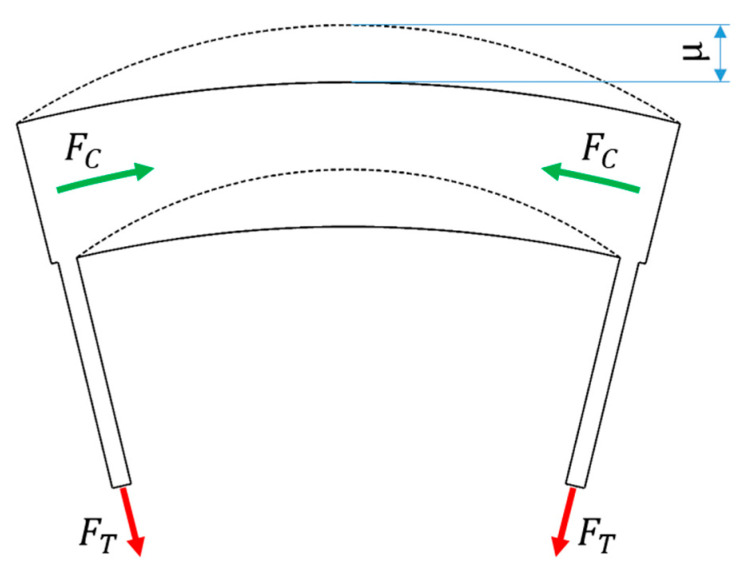
Buckling of the band segment due to tensile stresses on the NPT elastic structure (figure based on [23]).

The proper selection of materials and the method of making the shear beam/band and elastic structure will affect the smoothness of the rolling of the NPT [23]. Analyzing the band as individual beam segments supported at the ends by the elastic structure, it can be observed that the tensile stresses *F_T_* occurring in the elastic structure, resulting from the load on the NPT axle, cause the compression force F_C of the band segment (Figure 11). Exceeding the band compression capacity causes its radial displacement μ. The buckling of the band fragment (dashed line Figure 11) affects the non-uniformity of the wheel rolling radius value. The buckling value of the band fragment can be estimated using the formula [23]:

(3) $$\mu_{p/p}≅1.5\left(\frac{1-v^{2}}{El}\right)T{\left(\frac{r_{0}}{n}\right)}^{3}$$

- $\mu_{p/p}\;$—amplitude (peak to peak) of radial displacement (buckling) [mm],
- v—Poisson’s ratio of the shear beam/band material [-],
- E—Young’s modulus of elasticity of the shear beam/band material [N/mm^2^],
- l—geometric moment of inertia of the shear beam/band [mm^4^],
- $F_{T}$—spoke tension force [N],
- $r_{0}$—nominal radius [mm],
- n—number of spokes [-].

According to [23], smooth rolling is ensured when $\mu_{p/p}$ reaches small values and the following relationship is satisfied:

(4) $$\frac{r_{0}}{\mu_{p/p}}\geq 1500$$

**Figure 12:**
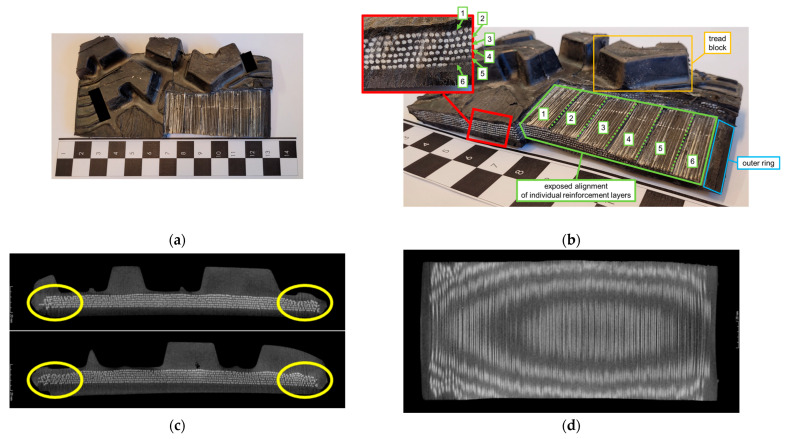
Portion of shear beam—NPT with 2D spoke: (**a**) view from the tread side with cut-outs, (**b**) identification: reinforcement layers; tread block; outer ring, (**c**) reinforcement layers (micro-computed tomography), (**d**) arrangement of the “threads” in the reinforcement layers (micro-computed tomography).

**Figure 13:**
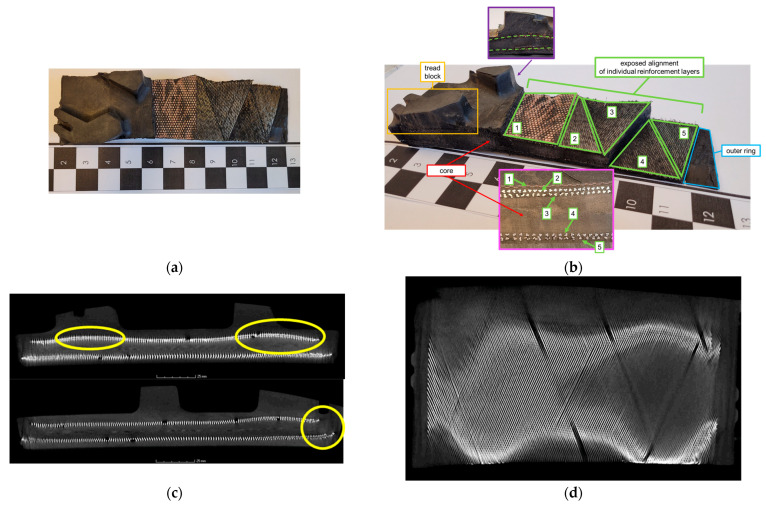
Portion of shear beam—NPT with 2D layered structure: (**a**) view from the tread side with cut-outs, (**b**) identification: reinforcement layers; tread block; outer ring, core, (**c**) reinforcement layers (micro-computed tomography), (**d**) arrangement of the “threads” in the reinforcement layers (micro-computed tomography).

Figure 12 and Figure 13 show shear beams that can be used in NPTs for Utility Terrain Vehicles (UTVs) and All Terrain Vehicles (ATVs) so that the information presented in patents and articles can be compared with actual NPTs. The NPT, a portion of which is shown in Figure 12, was equipped with 2D spokes. A view from the tread side with cut-outs exposing the reinforcement layers is shown in Figure 12a. The “threads” in each reinforcement layer are arranged parallel to the NPT plane. In Figure 12b, the outer ring (part of the elastic structure), six reinforcement layers, and tread block are colored blue, green, and orange, respectively. The core, which is located between the individual reinforcement layers (shown in zoom—a red rectangle), is difficult to separate (isolate)in the analyzed NPT. The elastomer between the reinforcement layers may result from the rubberization of the cord fabrics. The analysis of the shear beam structure was supplemented by tests using a Nikon/Metris XT H 225 ST micro-computed tomography scanner, located at the Laboratory of Materials Design and Rapid Manufacturing of Components at the Military University of Technologies (Faculty of Advanced Technology and Chemistry, Department of Advanced Materials and Technologies). The tests revealed a non-concentric arrangement of the reinforcement layers (especially on the NPT sides), which is marked by the yellow contour in Figure 12c, which presents photos from different locations of the tested portion. The reason may be due to the applied shear beam and tread production process, which allowed the layers to move. The arrangement of the reinforcement layers using micro-computed tomography is shown in Figure 12d, which confirms the parallel arrangement of the reinforcement layer “threads” in relation to the NPT plane.

The analysis of the NPT shear beam portion equipped with a 2D layered structure is shown in Figure 13. The cut-outs allowed the identification of 5 layers of outer (1, 2, 3 layers closer to the tread block) and inner reinforcement (4, 5 layers closer to the outer ring) and the core (Figure 13a,b). The cut-out and micro-computed tomography tests revealed the non-concentricity of the outer reinforcement layers in the immediate vicinity of the tread blocks (Figure 13b—purple rectangle) and the inner reinforcement layers on the shear beam sides (Figure 13c—yellow contour). The reason for the non-concentricity of the reinforcement layers is the manufacturing process. The cut-outs were necessary because one of the outer reinforcement layers (no. 1) was not revealed in the micro-computed tomography scans due to its different density. Reinforcement layers 2–5 (Figure 13b pink rectangle) are composed of three twisted “threads”. The diagonal arrangement of the reinforcement layers can be observed in Figure 13a,b,d. The core can be seen in the tested shear beam. Evaluating the effect of different shear beam designs on NPT characteristics is difficult due to the use of two different elastic structure designs by manufacturers. The materials used are also unknown.

Quick summary:

- The shear beam is intended to mimic the properties of compressed air used in pneumatic tires. It imitates the action of the bow, i.e., stores energy during bending.
- The use of a composite core makes it possible to meet expectations for low energy loss during deformation and low band thickness.
- The webbing geometry band ensures uniform pressure distribution in the contact zone and is characterized by simplicity of design (e.g., no need to use reinforcements) and manufacturing (e.g., centrifugal casting), while overcoming the limitations of the reinforced band, including the concentration of pressure at the beginning and end of the contact path and the need to use vulcanization.
- The band design should take into account and also result from the type of elastic structure intended to be used so that undesirable deformations (including buckling) do not occur.

Future research of shear beam and tread:

- The issue of aquaplaning is important in the context of vehicle traffic safety but is often omitted in NPT analyses. Ease of water drainage, e.g., through holes in the tread to the “inside” of the NPT, will effectively eliminate this problem, even with significant tread wear.

## 5. Materials Used in NPT

The authors of research papers on numerical studies of NPTs provide information on the parameters used in the constitutive models e.g., to describe the deformation energy of the elastomer. The selection of the model is preceded by experimental studies on the elastomer samples. In [5] an analysis of the literature on materials (tread compounds, frame-work materials, and support structure materials) used in NPTs was made. In the analysis of materials, patents are often omitted. One of the requirements for patents is that the invention must be industrially applicable. This means that the “industry” must be able to produce or use the claimed invention. This encourages some inventors to publish selected materials available on the market or to provide optimal ranges of material properties that allow the desired mechanical properties of the NPT to be realized. In the manufacturing process of pneumatic tires, the preparation of the green tire can take place in one or two stages. Difficulties in the confectioning of NPTs have necessitated the separation of the manufacture of individual components and the use of materials for selected NPTs’ parts that do not undergo the vulcanization process. A review of the literature shows that in the vast majority of cases, polyurethane is the most commonly used material, which is cast into a mold. Some references [24] suggest the use of injection or compression molding to produce an elastic structure. Based on the information in [21], the use of polyurethane is more environmentally friendly compared to traditional rubber tires because it generates less manufacturing waste and is easier to recycle. Another advantage of polyurethanes and non-rubber elastomers is that they can be used at low temperatures (environmental), resulting in reduced wear and increased fatigue life. Polyurethane has better abrasion resistance, is inert to other materials, and resists the oxidation that causes rubber to harden and crack. According to [21,24], in general, the following can be used: thermoplastic, cross-linked, or uncross-linked elastomer.

Based on a review of the patents, the following recommendations have been made for the NPT-specific parts:

- rim:
  ○ components made of aluminum or steel alloy,
  ○ using standard steel rim or one that has been modified to reduce the weight or to accommodate additional elements (e.g., sidewalls),
- elastic structure:
  ○ manufactured from a material that provides a tensile force in the part of the NPT above the axis and, at the same time, will provide bendability in the part of the NPT between the axis and the contact patch (‘top loader’ type NPTs), e.g., elastomers, steel cables, cord materials [21,23],
  ○ spokes material with a tensile modulus in the range of 10–100 MPa [23],
  ○ spokes are made of an elastomer with a Shore hardness of 40 D and a tensile modulus of approximately 21 MPa [21]—the hardness and tensile modulus requirements depend on the producing an elastic structure for the required load,
  ○ elastic cellular structure made of relatively hard material, e.g., with a Shore hardness of 80A–95A, 40D and a tensile modulus of 21–55 MPa [24,34],
  ○ elements of the cellular structure can have internal reinforcements of a material that will provide additional tensile strength, low compressive strength, high fatigue life, and the ability to bond to the elastomer, e.g., carbon fibers, Kevlar cord [21],
  ○ elastomer with a Shore hardness of 40–90 A [50],
  ○ varying hardness of the elastic structure achieved by changing the blend of cast elastomers during production [50] (another example of a gradient structure),
  ○ segmented elastic structure—a thermoplastic elastomer with Young’s modulus in the range of 45 MPa to 650 MPa, the elongation at break is greater than or equal to the yield strength, and preferably greater than 200% [8],
- shear beam—core:
  ○ material with an elastic modulus of 9–60 MPa [23], e.g., natural rubber, synthetic rubber, polyurethane, segmented copolyesters, and nylon block copolymers,
  ○ solid core—an elastomer with a dynamic shear modulus of 3–20 MPa [47],
  ○ composite core—elastomer layers with a modulus of approx. 3 MPa and approx. 30 MPa [47],
  ○ composite core—a ‘softer’ elastomer with an elongation at break greater than 180% (determined by Advancing Standards Transforming Markets—ASTM D412 at 100 °C) and a dynamic shear modulus of 1.5–5 MPa (determined by ASTM D5992); a ’harder’ elastomer with a dynamic shear modulus G at least three times greater than the ’softer’ elastomer [47],
- shear beam—reinforcement layers:
  ○ any material that meets the requirements for tensile stiffness, bending stiffness, and compressive buckling resistance of the annular band, e.g., materials suitable for use as tire carcass reinforcements in conventional tires [48].

Some patent applications cite commercially available materials for performance examples as follows

- elastic structure:
  ○ two-component polyurethane PR1664D (PRC-DeSoto International, Inc., CDP Division) [50],
  ○ cast elastomer from the Tadco S series with a Shore hardness of 70–99 A (TA Davies Company) [50],
  ○ natural rubber, styrene butadiene rubber, and polybutadiene rubber [50],
  ○ thermoplastic elastomer—ARNITEL PL 420H and ARNITEL PL461 (DSM Products) [8],
- shear beam:
  ○ polyurethane B836 VIBRATHANE (Chemtura) [22].

## 6. Conclusions

The main advantage of NPT is the lack of need to maintain compressed air, the properties of which require the use of appropriate materials and the shape of the NPT part. NPT can mimic the vertical load-carry mechanism used in pneumatic tires, which mainly depends on the way the rim is connected to the shear beam/band, i.e., the elastic structure used. With the same dimensions of NPTs (outer diameter, width), the top loader mechanism will allow the transfer of greater vertical loads than the NPT bottom loader. NPT can be equipped with simplified rims because they do not require tightness to maintain compressed air, as is the case with pneumatic tires. Rim modifications also allow for forming the resultant stiffness of the NPT and vibration damping. The paper also presents solutions enabling the mechanical connection of the NPT to the rim, which facilitates the process of replacing a damaged NPT part. The use of a mechanical connection of the elastic structure to the rim is an important direction of NPT development in the context of using structures with different characteristics or repairing a damaged elastic structure. The elastic structure of the NPT is the most characteristic part of the NPT, often visible from its side. It will have a decisive influence on the displacement of the NPT axle. The elastic structure can be built from various materials, not only elastomer. The 2D elastic structure is the most common NPT structure, which is characterized by low susceptibility to lateral deformation. A properly geometrically shaped 3D structure combined with the right selection of materials will provide lateral deformation at the required level. There is a noticeable increase in the use of bionic elastic structures that allow for the optimization of the structure in the context of increasing the load-bearing capacity and minimizing the weight of the NPT. The shear beam is intended to mimic the properties of compressed air in pneumatic tires. It imitates the action of the bow, i.e., stores energy. The elastic structure and the shear beam are the NPT parts that are responsible for the vertical load-carrying mechanism. The main share in transferring vertical load is held by the part that is characterized by higher stiffness. In order to provide a high load capacity (top loader type), it is necessary to provide a susceptibility to buckling/deformation as a result of compression under the wheel axle. In the described case, the shear beam mainly transfers vertical loads.

Shear beam analysis of two NPTs revealed different materials were used as reinforcements. The reinforcement layers were placed at different angles to the wheel’s center plane. Their influence on the movement properties (especially in curvilinear movement) is not known. The use of the vulcanization process may cause difficulties with the correct (designed) location of the reinforcement layers.

The use of webbing geometry significantly facilitates the production of the band in comparison to reinforced composite shear beams while maintaining similar characteristics.

The use of bionic structures has been successfully applied in a flexible structure. Another promising area is the shear beam (optimization of mass and strength), but no such solutions were found in the review.

Articles presenting the results of simulation studies only provide information on the coefficients of the material models used. Another approach can be found in patents (often omitted in literature reviews), where optimal ranges of selected material properties of the invention are often presented, as well as the trade names of products from which the NPT component was made.

## Data Availability

No new data were created or analyzed in this study. Data sharing is not applicable to this article.
